# Advances in Transdermal Drug Delivery Systems: A Bibliometric and Patent Analysis

**DOI:** 10.3390/pharmaceutics15122762

**Published:** 2023-12-12

**Authors:** Aniello Cammarano, Stefania Dello Iacono, Caterina Meglio, Luigi Nicolais

**Affiliations:** 1 Materias Srl, Corso N. Protopisani 50, 80146 Naples, Italy; 2 Institute of Polymers, Composites and Biomaterials (IPCB), National Research Council, P.le Enrico Fermi 1, 80055 Portici, Italy

**Keywords:** transdermal drug delivery, patent analysis, bibliometric analysis, transdermal administration, controlled release

## Abstract

Transdermal drug delivery systems have become an intriguing research topic in healthcare technology and one of the most frequently developed pharmaceutical products in the global market. In recent years, researchers and pharmaceutical companies have made significant progress in developing new solutions in the field. This study sheds light on current trends, collaboration patterns, research hotspots, and emerging frontiers of transdermal drug delivery. Herein, a bibliometric and patent analysis of data recovered from Scopus and The Lens databases, respectively, is reported over the last 20 years. From 2000 to 2022, the annual global publications increased from 131 in 2000 to 659 in 2022. Researchers in the United States, China, and India produced the highest number of publications. Likewise, most patent applications have been filed in the USA, China, and Europe. The recovered patents are 7275, grouped into 2997 patent families, of which 314 were granted. This study could support the work of decision-makers, scientific managers, or scientists to create new business opportunities or save money, time, and intellectual capital, thereby defining when a research or technology project should be a priority or not.

## 1. Introduction

Transdermal drug delivery (TDD) represents an alternative drug administration to oral delivery or hypodermic injection [1,2,3]. Since ancient times, people have placed substances or active molecules on the skin for their therapeutic effects, and in modern times, a variety of topical preparations have been developed to treat local medical conditions. In 1979, the first transdermal system for systemic delivery, treating motion sickness, was approved in the United States. Later, other drugs were approved for TDD (nicotine, estradiol, fentanyl, lidocaine, and testosterone), and different delivery systems (iontophoretic, ultrasonic, gels, patches, microneedles) were developed.

Aside from the oral route, TDD has several advantages [4] over hypodermic injections, which are invasive, painful, generate medical waste, pose a risk of disease transmission by needle reuse, especially in developing countries [5], and cannot be self-administered. TDD improves patient compliance and can provide a time-controlled release; it becomes especially important when drugs could be prematurely metabolized by the liver. Furthermore, these systems are generally inexpensive. However, high-molecular-weight drugs face significant limits in successful delivery due to the stratum corneum (SC). Therefore, several technologies have been developed to enhance the permeability of drugs across the SC, including chemical penetration enhancers and physical and electrical enhancement approaches, such as thermal ablation, electroporation, ultrasound, jet injection, and microneedles. These technologies provide a painless and controlled release of anti-inflammatories, vaccines, insulin, lidocaine, and other drugs. Therefore, due to the impact of the TDD system on patients’ health, research and the development of new knowledge on these represent an urgent topic. In a state-of-the-art technological environment where convergence and dissemination are accelerated, and the search for future technologies is an important issue, scientific and patent analysis is emerging as a central element. The bibliographic and patent scenario study in TDD can provide insights into the progress of technologies and knowledge involved in improving the patient’s life and help assess their use strategy. By analyzing both papers and patents, researchers can make well-informed decisions about target selection and priority.

Patents have long been regarded as essential incentives to promote innovation due to the long, costly, and risky nature of the research and development process, particularly in the field of new drugs and delivery systems. The problem is further compounded by current statistics indicating a decrease in the number of novel breakthrough drugs [6] despite the alleged surge in investments into pharmaceutical R&D. On the other hand, the number of innovative delivery systems of existing medicines is growing, demonstrating that pharmaceutical companies have been increasingly focusing their research on new devices, rather than on new drugs or other revolutionary innovations. Pharmaceutic companies invest in new drug delivery systems, including TDD, to extend products’ profitable lifecycles and provide patients with improved medications [7], guaranteeing competitive and financial advantages by administering drugs with innovative therapeutic benefits.

This paper presents an overview of bibliography and patents produced in academia and industry through bibliometric and patent analysis. Bibliometric analysis can help identify trends in research, the journals publishing the most articles on this topic, and the countries producing the most research in this area. Patent analysis may allow the identification of stakeholders in TDD technologies and types of products or solutions being developed for the market. By comparing the two analyses, researchers and companies could collaborate in this field to develop new products. It is expected that this study could support the work of decision-makers, scientific managers, or scientists to create new business opportunities or save money, time, and intellectual capital and define when a research or technology project should be a priority or not. This work begins with a focus on the scientific production of TDD, followed by an analysis of patented technologies in the last 22 years (2000–2022). The analysis has been developed, including co-occurrence and network analysis, to summarize the progress in TDD and detect the hotspots or emerging trends and contributions of authors, journals, institutes, or countries using a specific data sample. The software VOSviewer (version 1.6.19) has been used to develop a visual map of the co-occurrence between keywords and researchers. Furthermore, a network analysis based on the presence of keywords in papers and patents is developed. This analysis assumes that patents that share similar keywords are related in some way, and these relationships can be used to identify trends in innovation.

The work is a bibliometric and patent analysis focused on tracking the development and evolution of transdermal drug delivery systems. Even if the bibliometric review was conducted with a wide and impartial scope, its limits are evidently due to a unique source of documents. In fact, despite the SCOPUS’ status as a frequently used, respected, and extended database [8,9], it inevitably omits some publications. Furthermore, this study lacks a deep evaluation of the goodness of the bibliography, giving equal weight to publications over the whole quality spectrum. Similarly, there are also some limitations in patent analysis because documents were recovered only from the database that LENS can access.

## 2. Materials and Methods

### 2.1. Bibliometric Data Collection and Analysis

**Figure 1:**
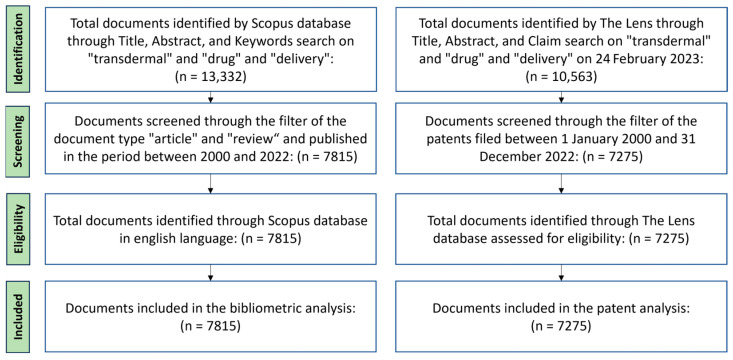
PRISMA flow diagram for data collection by SCOPUS e LENS.

In this transversal study, the publication’s data were collected in a single day (24 February 2023) and downloaded as a csv file from Scopus (www.scopus.com). Information clustering and the outputs were performed with Excel’s programming functions. The proposed method is based on programming scripts to automatically obtain bibliographic data from scientific publications using the free Scopus Database API Interface, Scopus being the largest database of peer-reviewed literature in different scientific fields. The data sample development strategy is shown in Figure 1. The search terms were determined by the query ((TITLE-ABS-KEY(transdermal AND drug* AND delivery) AND PUBYEAR > 1999 AND PUBYEAR < 2023) AND (LIMIT-TO (DOCTYPE,”ar”))). Refining the search to articles (document type) published in the period between 2000 and 2022, information was collected, such as publications, authors, countries, institutions, founding sponsors, journals, keywords, and citations.

The recorded data were also processed using bibliometric visualization software to extract and analyze publication data and create knowledge maps [10]. In the current study, we used VOSviewer (www.vosviewer.com) version 1.6.19 to obtain bibliographic information on researchers, research institutions, countries, citations, and keywords from Scopus csv files and to create network maps. Co-authorship and co-occurrence analysis were performed to identify the key themes and investigate hotspots in the literature. Co-authorship analysis reveals patterns of collaboration among countries [11]. The coexistence of keywords analysis uses the frequency of multiple words to identify their proximity, thus demonstrating hot topics and trends in the discipline. In this study, the top high-frequency keywords appearing in the retrieved publications were analyzed to explore hotspots in the TDD research. In VOSviewer visualization maps, each node is represented by a labeled circle. Larger circles indicate a higher frequency in the co-occurrence analysis. The color of each circle is specific to the cluster to which it belongs. The thickness and length of links represent the strength of the connection between the corresponding nodes. A maximum of 1000 lines have been set to display the 1000 most robust links. VOSviewer was used to identify the cluster and the relationships among these clusters by the colored map [12] and to indicate the keywords that are covered by a cluster. Cluster interpretation may indicate trends and patterns in the research literature. By analyzing clusters of related keywords, researchers can gain insights into the most active research fields and identify potential areas of collaboration.

### 2.2. Patent Data Collection

On 24 February 2023, a patents search was performed by using The Lens database (www.lens.org), with the following search string filling in the “Query Text Editor” field: (title:(transdermal) OR abstract:(transdermal) OR claim:(transdermal)) AND (title:(drug*) OR abstract:(drug*) OR claim:(drug*)) AND (title:(delivery) OR abstract:(delivery) OR claim:(delivery)). The Lens is a friendly database that accesses Espacenet, USPTO, WIPO, and Australian patent databases, being a broad-spectrum information retrieval tool. The search was limited to patents filed in the period between 1 January 2000 and 31 December 2022. The growth of TDD-related patents in 2000–2022 was analyzed, and the key origins (country), key owners, and classifications were examined. In addition, a network analysis based on keyword occurrence in patents has been developed. This analysis assumes that patents that share similar keywords are related. Clustering and information outputs were performed using Excel, while the network analysis based on keyword occurrence of patents was developed with VOSviewer.

## 3. Results and Discussion

### 3.1. Annual Global Publication on Transdermal Drug Delivery

**Figure 2:**
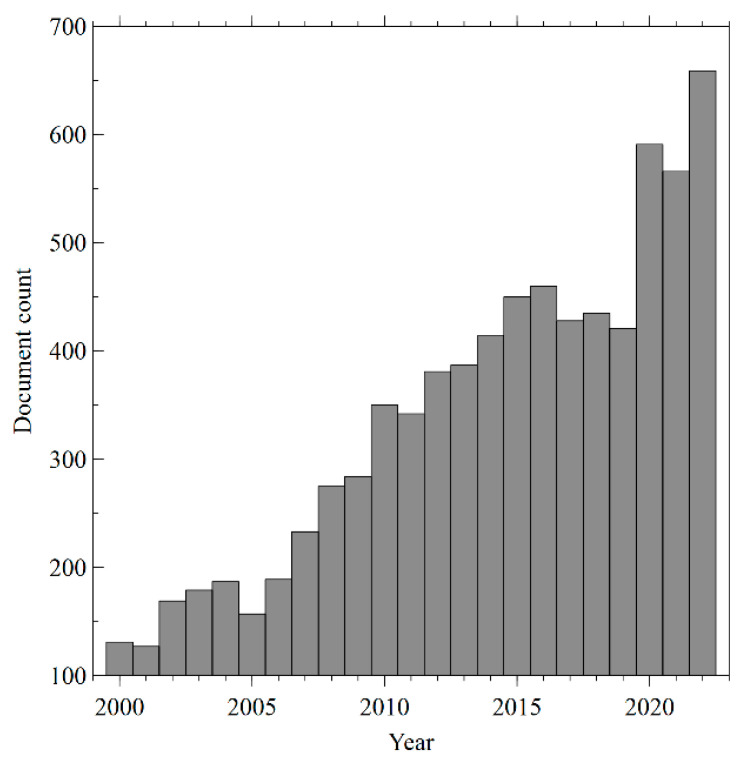
Number of papers per year.

A total of 7815 articles related to transdermal drug delivery research from 2000 to 2022 were recovered from Scopus. The production of annual publications on TDD is shown in Figure 2. Global annual publications increased from 131 in 2000 to 659 in 2022, with an average annual growth rate of 6.4% and a maximum increase in 2020 (+28.8%), probably because of the global pandemic emergency. In response to the global health emergency, the scientific community has reallocated research programs to find innovative solutions and prevent and fight COVID-19. The collected data support the hypothesis that this crisis has induced a sudden increase in research output in the areas of biomedical research, including TDD.

In contrast, in some years, there was a stagnation in the number of papers published. The temporary lack of funding for R&D expenditure could explain this phenomenon. Research can be costly, and without adequate financial resources, it can be difficult for researchers to carry out their work. For example, in the 2010s, after the global financial crisis triggered by the bankruptcy of Brother Lehman Bank (2008), the growth of international production stopped, and this was first reflected in trade. Worldwide exports of goods and services, which had grown for decades, slowed down significantly relative to economic growth [13]. In the following years, the United States, Ireland, Spain, Greece, and many other European countries went into a deep recession. Driven by the economic downturn, global R&D spending fell [14]. This has led to fewer scientific findings and a decrease in the number of scientific publications. On the other hand, in some years, an increase in the number of scientific articles could be related to the approval of new active molecules for TDD by the US Food and Drug Administration (FDA). The acceptance process for new drugs involves extensive research and testing to prove their safety and effectiveness. This research often leads to the production of new scientific knowledge, which can be disseminated through scientific products. In addition, the approval of a new drug by the FDA can generate interest and further research in related areas, contributing to the increase in the number of publications. For example, in 2006, three drugs were accepted by the FDA: Fentanyl HCl (Alza), Methylphenidate (Shire), and Selegiline (Bristol-Myers Squibb) [15]. Such approvals could have contributed to an increase in the publications in TDD in the subsequent years. The same phenomenon has been recorded when other active molecules for transdermal release have been approved [16,17].

### 3.2. Top 10 Journals and Cited Articles

The retrieved articles on TDD were published in 160 journals. Table 1 lists the top 10 journals that published the most articles on TDD, accounting for 26.5% of the total publications. The International Journal of Pharmaceutics was the most productive journal (525 publications) and the most highly cited journal (23,596 citations), followed by the Journal of Controlled Release with 262 publications and the Journal of Pharmaceutical Sciences with 226 publications.

Table 2 lists the top 10 most cited articles on TDD. “Biomedical applications of collagen” by Lee et al., published in the International Journal of Pharmaceutics in 2001 [18], was the most cited article (1516 citations), followed by “Penetration enhancers” by Williams et al., published in Advanced Drug Delivery Reviews [19]. A total of 6995 articles were cited 208,512 times, with a median number of 29.8 citations.

Table 3 reports the top 10 authors who published papers on TDD and their related affiliations.

**Figure 3:**
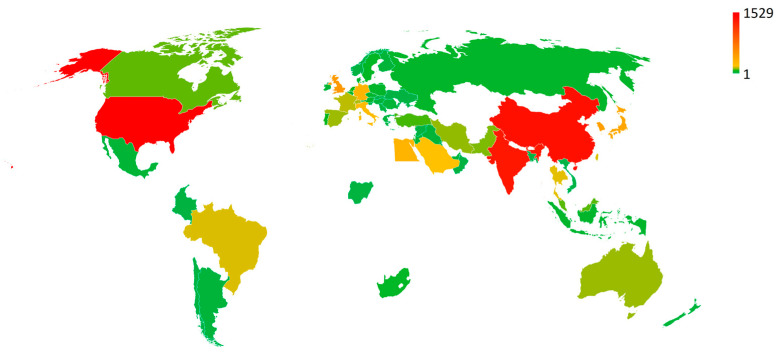
World areas colored according to the number of publications on TDD: green for the less numerous and red for the more abundant.

In terms of the absolute number of publications on TDD, researchers in the United States, China, and India produced the highest number [28]. In Figure 3, the world areas in which the publication on TDD is most abundant are colored in red.

Table 4 shows the top 10 funders, such as government agencies or non-profit organizations. By investing public funds in TDD research, the US and China have contributed significantly to the publication of scientific papers in this field. In the United States, funding comes from organizations like the National Institutes of Health (NIH), the National Institute of Biomedical Imaging and Bioengineering, and the National Science Foundation (NSF). In China, funding hails from agencies like the National Natural Science Foundation of China (NSFC) and the Ministry of Science and Technology (MOST).

The main subject areas derived from a bibliographic analysis on TDD (i.e., pharmacology, medicines, biochemistry, chemistry, and material science) correspond to the issue of research activities carried out in this field (Table 5).

### 3.3. Co-Authorship of Countries

**Figure 4:**
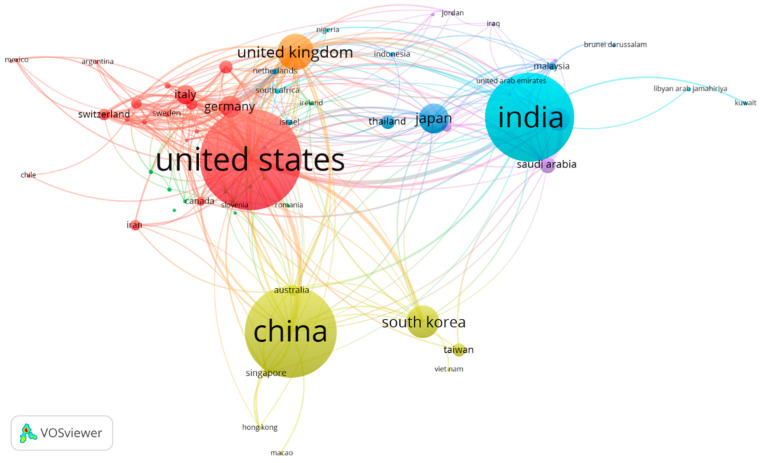
The co-authorship network in TDD studies, including 65 countries, grouped into 8 differently colored clusters.

It is widely recognized that there is a great interest in health research, and TDD is one of the most appealing areas. The results showed that a total of 100 countries contributed to TDD research, as shown in Figure 3. USA (1529 publications) is the most productive country, followed by China (1371), India (1325), United Kingdom (486), South Korea (439), Japan (396), Egypt (297), Germany (290), Italy (248), and Saudi Arabia (195). VOSviewer was used for co-authored country analysis to show links of international collaborations on TDD (Figure 4). In the network visualization, countries are represented by circles; for some minor countries, the symbol cannot be shown to avoid overlapping. The 1000 strongest links are displayed by lines. In this visualization, the distance between two countries approximately indicates their relatedness in terms of co-authorship links. The closer two countries are located to each other, the stronger their relatedness.

The network, including 65 countries, was defined by 8 differently colored clusters connected through co-authorship links. The largest cluster (red), consisting of 19 countries, 3275 articles, and 117,835 citations, is centered on the United States, Germany, Italy, and France. The US has the most significant number of cooperating partners (51). China, Australia, and South Korea are part of the second largest cluster (green), consisting of 8 countries, 3240 articles, and 51,557 citations. India is the center of the blue-sky cluster (third in size), consisting of 5 countries, 1420 articles, and 28,195 citations.

### 3.4. The Co-Occurrence Analysis of the Top Keywords

Co-occurrences indicate the number of documents in which a keyword occurs. Keywords in research papers define the research topic and are used to make the scientific article more detectable. In this study, VOSviewer extracted and clustered the top 92 keywords, as shown in Table 6.

**Figure 5:**
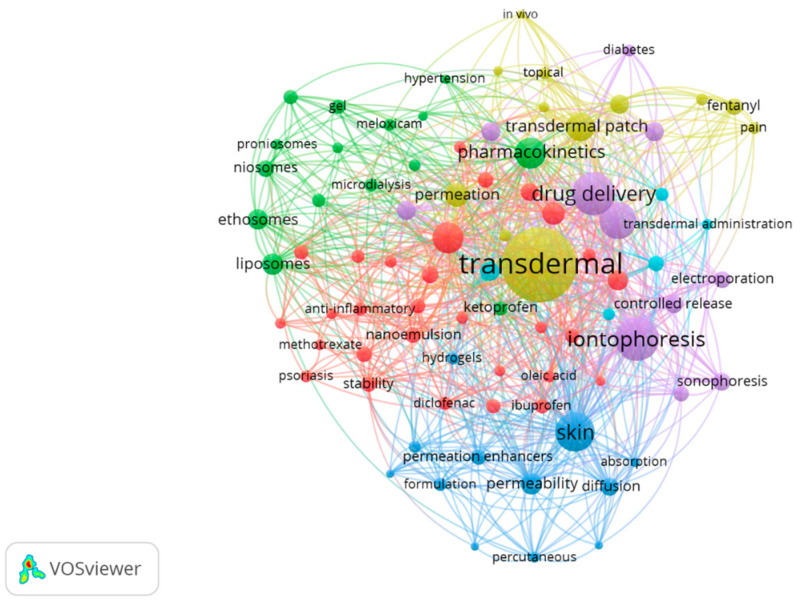
The co-occurrence cluster analysis of the top keywords in TDD papers. The size of the circle depends on the keyword frequency. All keywords are grouped into differently colored six main clusters.

Figure 5 shows a network map of the top keywords in six clusters with their co-occurrence, represented by a circle. The size of the circle is determined by the frequency of the keyword. Lines between keywords represent links, of which the strongest 1000 are displayed. The keywords transdermal (958), iontophoresis (398), drug delivery (379), skin (332), and microneedle (295) are placed at the center of the network. All keywords were grouped into six main clusters and displayed by red (cluster 1), green (cluster 2), blue (cluster 3), yellow (cluster 4), purple (cluster 5) and blue-sky (cluster 6) circles.

Cluster 1—in red—represents different studies that examined the skin absorption of commercially available topical nonsteroidal anti-inflammatory drugs (NSAIDs, e.g., diclofenac, ibuprofen) [29,30], modified formulations to obtain superior anti-inflammatory activity [31]. The therapeutic effect of NSAIDs depends on the drug’s ability to penetrate and permeate the skin [32]. Recent studies have investigated the use of transdermal delivery of NSAIDs for the treatment of inflammatory diseases, such as rheumatoid arthritis and osteoarthritis [33,34]. Studies have shown that transdermal delivery of NSAIDs could offer several advantages over oral administration, including improved bioavailability, reduced systemic exposure, and lower risk of gastrointestinal adverse effects [35]. Other recent studies have investigated the use of transdermal delivery of anti-inflammatory molecules for the treatment of psoriasis [36]. The studies demonstrated that the transdermal delivery of the biological molecules could reduce inflammation and improve skin barrier function [37].

Cluster 2—in green—mainly represents the TDD application in pain management [38]. Fentanyl and buprenorphine are opioid analgesics that are commonly used to treat moderate to severe pain [39]. Both drugs have a high potency, which means that they can be effective at lower doses than other opioids [40]. Transdermal administration of these drugs may provide a more consistent and predictable delivery than oral administration, which may lead to better pain control and fewer side effects. Lidocaine is a local anesthetic that can be used to relieve pain in a specific area [41]. Transdermal delivery of lidocaine has been investigated for the treatment of chronic pain conditions such as neuropathic pain and post-herpetic neuralgia. Lidocaine patches can provide long-lasting pain relief with fewer side effects compared to systemic administration. Estradiol is a hormone that has been investigated for the treatment of menopausal symptoms such as hot flashes. However, it has also been studied for its potential analgesic effects in the treatment of pain [42]. Transdermal delivery of estradiol can provide a sustained release of the drug, which may lead to improved pain control and fewer side effects than oral administration. The design of transdermal drug delivery systems and the factors affecting drug permeation, bioavailability, and pharmacokinetics are important considerations in the development of these systems [43].

Cluster 3—in blue—represents the papers on the importance of skin permeability, diffusion, and absorption in transdermal drug delivery, as well as the use of permeation enhancers, hydrogels, and other formulations to improve drug delivery. They also provide insights into mathematical models and strategies for enhancing drug solubility and permeability. For example, Mitragotri et al. describe mathematical models used to predict drug permeation across the skin [44], including factors such as solubility, diffusion, and permeability enhancers. Prausnitz et al. discuss the challenges of providing proteins through the skin [45] and various strategies to improve their absorption, including the use of permeation enhancers, hydrogels, and other formulations. Williams et al. examine various classes of permeation enhancers [19] used to improve drug absorption through the skin, including surfactants, solvents, and other chemicals. In addition, vesicular systems are studied to be used to deliver vaccines through the skin, which is a promising route for vaccination due to the abundance of antigen-presenting cells in the skin [46]. For example, niosomes have been used to administer hepatitis B vaccine [47]. Vesicular systems are also studied for gene delivery through the skin, which is a non-invasive and painless approach to gene therapy [48]. For example, liposomes have been used to deliver siRNA for the treatment of skin diseases such as psoriasis.

Cluster 4—in yellow—represents some studies on the development of nanoparticles as a delivery system for TDD applications. In this research, chitosan plays an important role [49] for TDD due to its unique properties, such as biocompatibility, biodegradability, and mucoadhesiveness. Chitosan nanoparticles have several advantages in transdermal drug delivery, such as improved drug permeation and bioavailability, prolonged drug release, and reduced skin irritation. Nanoparticles can also protect the drug from degradation and enhance its stability. Additionally, chitosan nanoparticles can be functionalized with various molecules, such as permeation enhancers and targeting ligands, to further improve their efficacy in TDD. Several studies have investigated the use of chitosan nanoparticles for transdermal delivery of various drugs, including antihypertensives [50], anti-inflammatory agents, and anticancer drugs [51]. Also, in vitro and in vivo studies have demonstrated the potential of chitosan nanoparticles for transdermal delivery of these drugs [52], showing improved permeation and bioavailability compared to conventional formulations. However, there are still some challenges associated with the use of chitosan nanoparticles in TDD, such as poor stability under certain conditions, low drug loading capacity, and potential toxicity issues [49].

Cluster 5—in purple—represents methods and techniques to optimize the drug delivery of micro and nanoparticles into the skin. The keywords of this cluster, iontophoresis [53], microneedles [20], sonophoresis [54], electroporation [55], and ultrasound [56], are all techniques that can be used to enhance TDD, including drugs such as insulin [57] used to treat diabetes. The combination of these techniques can further increase drug permeation and effectiveness and may offer potential alternatives to traditional injection-based therapies.

Cluster 6—in blue-sky—mainly represents applications of vesicular delivery systems as carriers in TDD applications [58]. Different types of vesicular drug delivery systems have been reported as keywords, such as bilosomes, pharmacosomes, emulsomes, transfersomes, liposomes, and niosomes. Vesicular delivery systems are widely used as carriers in TDD due to their ability to enhance drug penetration across the skin. Recovered studies report that these systems can improve drug solubility, stability, and bioavailability, as well as reduce systemic toxicity and increase therapeutic efficacy. In general, liposomes are spherical structures composed of a lipid bilayer that can encapsulate hydrophilic or hydrophobic drugs. Niosomes are like liposomes but are composed of non-ionic surfactants. Transfersomes are highly deformable lipid vesicles that can penetrate the SC of the skin more efficiently than liposomes and niosomes. For example, transfersomes have been used to deliver hormones [59], such as testosterone and estradiol, for hormone replacement therapy.

In Table 7, some examples of articles included in the above-mentioned clusters are reported.

### 3.5. Patent Database Analysis

The total number of recovered patents includes 7275 documents grouped into 2997 patent families, of which 314 were granted. A total of 110 distinct applicants were found. In the retrieved database, 152 documents were cited more than 100 times (highly cited patents). The number of citations measures the importance of a patent, and highly cited patents are precursors that best define the state of the art, indicating the level of influence that a patent has had on subsequent inventions. The indexing citation is an important tool for evaluating the value and impact of a patent. Patents with high citation indexes are more likely to be licensed or sold at a higher price, as they are seen as more innovative and influential in their field. The document US 8 617 071 B2, “Analyte monitoring device and methods of use” [71], with 1197 citations, was the most cited by the other patent documents. This patent, filed in 2000 and owned by Abbott Diabetes Care Inc. (Alameda, CA, USA) describes an electrochemical sensor-based device for analyte monitoring that is also part of a TDD system to alter the analyte level based on the data obtained using the sensor. It is important to stress that technologies considered essential could be mentioned more times. In addition, older patents are cited more often simply because they have had more opportunities to be cited, while new applications could take more time to be found out by other players and be cited. By understanding the level of influence and impact of a specific patent, businesses and researchers can make informed decisions about how to invest their resources and develop research activities.

Since the World Intellectual Property Organization (WIPO) application is related to an undefined place for protection, these results will be excluded from the potential markets for the exploitation of technologies analysis. According to Table 8, which reports the top 10 countries in which patents have been filed or granted, the US was the preferred location to apply technologies related to TDD. The high number of biotech companies could be a substantial factor influencing applicants’ interest. China, according to Table 8, is still a place of considerable interest for patent applicants on TDD. The Chinese patent office was preferred over the European Patent Office.

**Figure 6:**
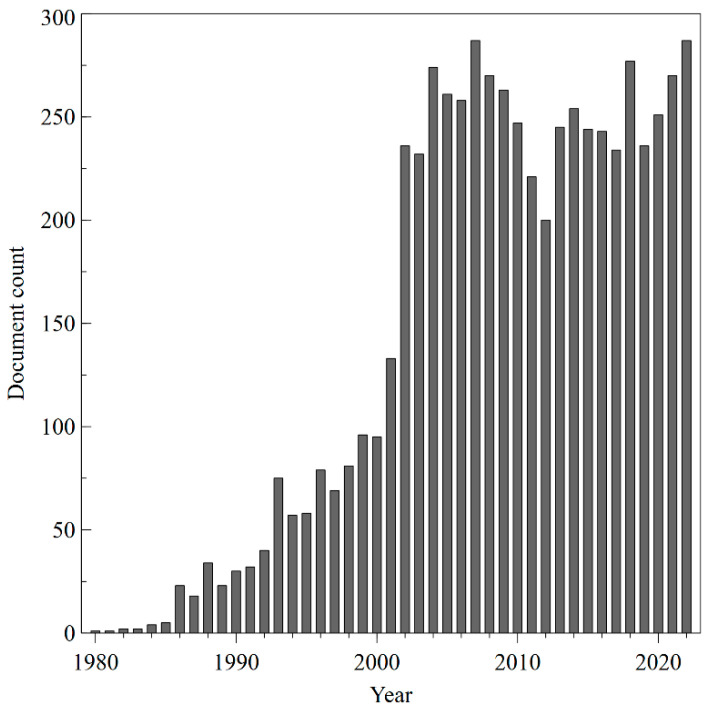
The number of TDD patent applications in the years.

The evolution of the number of patent applications through the years is shown in Figure 6, in which a longer time interval than that concerning the study of interest is examined to consider the trend in its wholeness.

One way to analyze this growth of patent applications is by an *S*-curve. Expanding the time range of the number of applications from 1980 to 2022, the *S*-curve shows three stages: an initial slow growth phase, followed by a rapid acceleration phase, and finally, a saturation phase in which growth stabilizes [72]. This graph represents the growth of TDD technology and the rate at which new solutions are invented and patented. During the early stages of technological development, the growth of patent applications was slow due to the rarity of new technologies and the limited innovation background. However, as more and more individuals and organizations focus on developing new technologies, the number of patent applications gradually increases, leading to the acceleration phase of the *S*-curve. At this stage, the rate of growth of patent applications noses up as more people and companies apply for patents to protect their inventions. The acceleration phase is characterized by a sharp increase in the rate of change in patent applications (2002–2004). Eventually, the growth rate of patent applications reaches a plateau during the saturation phase as most of the new technologies have been invented, causing a slowdown in the rate of innovation. This plateau was achieved in the 2004–2020 period. Considering that patent applications are usually published 18 months after the filing date, the number of applications in the last year and a half of the analyzed period (2021–2022) could be underestimated in Figure 6.

**Figure 7:**
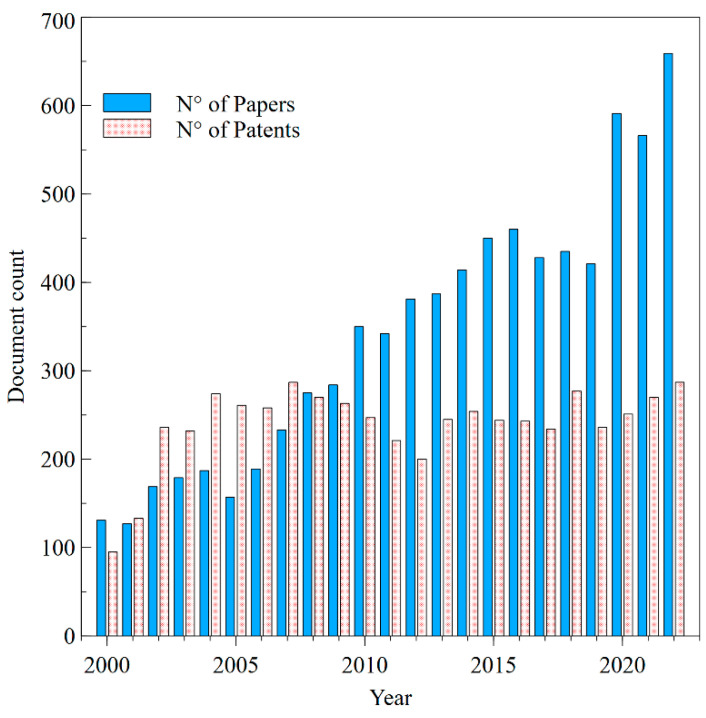
Scientific papers and patents production on TDD across the years.

Focusing on the time interval of interest for the analysis and directly comparing the number of publications and patents per year, as shown in Figure 7, it should be noted that the number of articles is growing monotonically, while the number of patents has reached a constant value over the last years.

As shown in Table 9, the most common Cooperative Patent Classification (CPC), A61K, concerns “devices or methods specially adapted for bringing pharmaceutical products into particular physical or administering forms” and “materials for disinfection or sterilization, or for bandages, dressings, absorbent pads.” These CPCs adequately describe the retrieved patent database.

The ten most active patent applicants are listed in Table 10, where United States companies are the major players in the patenting activities of TDD technologies.

The low presence of universities in the top ten applicants probably indicates that most of the TDD technologies are mature enough to be fully explored by companies. Often, academic patents are filed for academic purposes and not for commercial reasons [73,74]. A very singular case widely studied is that of microneedles technology [75], which has generated numerous papers, patents, and clinical studies. The field of microneedles is widely patented by universities, and it has excellent potential in the transdermal delivery of innovative products to improve health and quality of life.

Another intriguing aspect observed when examining patents is that, despite a substantial increase in the number of research publications related to TDD in India, there has been a relatively low number of patents filed in this country. This phenomenon may be due to a lack of resources and expertise in patent filing among Indian researchers.

Noven Pharmaceuticals (NP), the first TDD patent candidate, is a Miami-based pharmaceutical company specializing in the development and marketing of innovative therapies for central nervous system (CNS) disorders. As shown in Table 10, the company has a strong patent strategy to protect its intellectual property (IP) and maintain its competitive edge in the market. NP’s patent portfolio includes a mix of both product and process patents. This allows the company to protect its drug products and the manufacturing processes and formulations used to produce them. The company’s IP resources are focused on its core areas of expertise, including CNS disorders therapies. NP has also partnered with various pharmaceutical companies to develop and market TDD systems. Some of the commercial products that have been developed using NP drug delivery technologies include: (i) Daytrana—a transdermal patch for the treatment of attention deficit hyperactivity disorder (ADHD), developed in association with Shire Pharmaceuticals (ii) Minivelle—a transdermal patch for the treatment of menopausal symptoms, developed in collaboration with Novartis Pharmaceuticals (iii) Butrans—a transdermal patch for the treatment of chronic pain, developed in partnership with Purdue Pharma. In Table 10, Alza Corporation (AC) is in second place. AC was a private biopharmaceutical company specializing in the development of TDD technologies that has filed patent applications for a few pertinent technologies and methods, including patches, gels, and other delivery systems. Furthermore, it has developed and patented some technologies related to (i) microneedle patches, (ii) transdermal gel formulations, and (iii) controlled-release transdermal patches. In 2001, Alza Corporation was acquired by Johnson & Johnson.

### 3.6. The Co-Occurrence Analysis of the Top Keywords of Recovered Patents

**Figure 8:**
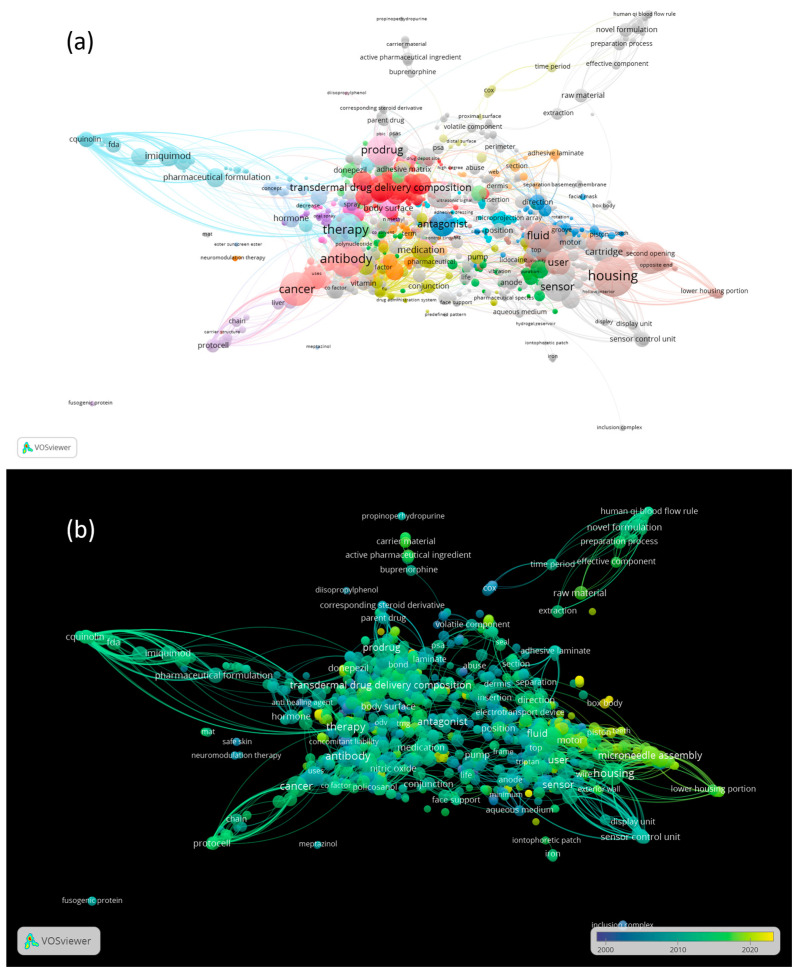
(**a**) Total strength of the co-occurrence links between keywords in TDD patents; (**b**) co-occurrence cluster analysis of the top of TDD patents keywords over time.

The correlation of keywords was calculated by counting the frequency of their occurrence together in the titles and patent abstracts. A co-occurrence network was created using VOSviewer. For each keyword, the total strength of the co-occurrence links with the other ones was evaluated (Figure 8a). The overlay visualization, shown in Figure 8b, differs graphically from the network view (Figure 8a) for differently colored keywords. In Figure 8b, the color of a keyword is determined by the patent application date that contains it: colors range from blue (older patents) to green to yellow (newer patents). In the overlay visualization, the latest yellow-colored patents, for example, are related to the area of microneedle technologies.

Microneedle technologies have been the subject of academic and industrial researchers’ intensive research and development efforts. Thus, the number of patents describing microneedles as novel minimally invasive devices for drug delivery purposes has grown exponentially in recent years [76].

**Figure 9:**
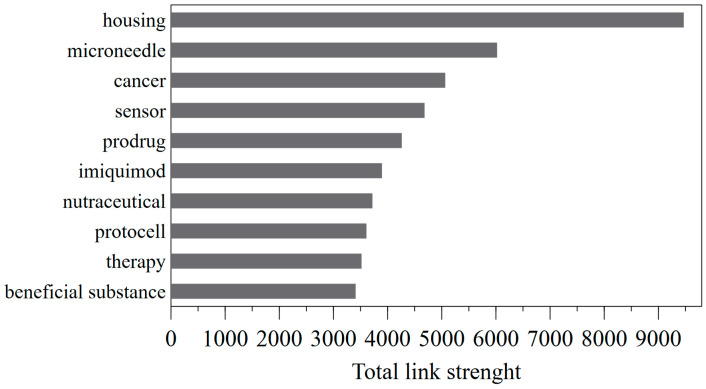
Total link strength of top ten keywords in the title/abstract/keyword of the recovered patents.

In Figure 9, keywords with the highest total link strength have been selected and reported. The total link strength obtained from co-occurrence networks indicates the number of patents in which two keywords occur together. Here, a stronger link typically indicates a higher frequency of the two keywords appearing together in patents.

Analyzing the keywords with the highest total link strength, the main topics of patents on TDD applications include housing (9469) [15], microneedle (6022), cancer (5065), sensor (4683), prodrug (4262), imiquimod (3896), nutraceutical (3720), protocell (3610), therapy (3519), and beneficial substance (3410). These keywords are placed at the center of the network, represented in Figure 8a. The closer the keywords are to each other, the stronger their relationship is. The keywords housing, sensor, and microneedle, which are close to each other in the network, refer to medical devices for TDD. Some patents describe a transdermal drug delivery device that includes a housing to contain a reservoir of drugs and a microneedle array for drug delivery through the skin. Table 11 lists the most cited active patents that refer to a medical device, reporting information on active patent applications filed in the last 20 years, including the application number, application date, title, and owners. The device can also include a sensor, such as a biosensor, to monitor drug levels in real-time and ensure accurate dosing. The sensor may be integrated into the housing or positioned near the microneedle array for efficient monitoring. Furthermore, the device can also include a controller, which may be in the housing or in a separate unit, to adjust the drug administration according to the sensor readings. This combination of a microneedle array, drug reservoir, sensor, and controller in one housing provides a convenient and effective TDD system for various medical issues.

A list of the top cited patents that refer to TDD solutions for cancer treatment and administration of prodrugs are reported in Table 12 and Table 13, respectively.

## 4. Conclusions

This paper proposes an overview of the last twenty years of publications and patents about TDD to identify and compare the main functions, application fields, and technological aspects. The main novelty concerns the use of both papers and patents to provide a comparative perspective on how the frontier of knowledge is advancing in the field of TDD. The distribution of scientific papers is growing constantly, while the number of patents filed per year is constant. This is because the scientific community has invested more resources in the research and study of TDD solutions, leading to more publications. Research in this field has moved towards developing new formulations and technologies capable of overcoming the limitations of the transdermal delivery route. However, these studies may not necessarily involve the submission of new patents, as some of the innovations may not meet the patentability criteria or may not be considered commercially viable. Moreover, scientific publications could be considered prior art, which means that they can prevent the patentability of subsequent inventions that are not sufficiently different from them. Additionally, some of the research in the TDD field focuses on fundamental scientific understanding rather than practical applications. Furthermore, the patent landscape for TDD has become more crowded, making it more difficult to patent.

Despite a partial overlap between technological trends in the TDD developed in scientific articles and those reported in the patent, bibliometric and patent analyses show some important differences. In scientific papers, key technological trends in TDD include (i) the development of new materials and formulations that improve drug permeation and skin penetration, such as lipid-based systems and hydrogels (cluster 1, cluster 3, cluster 4, cluster 6); (ii) the development of physical methods to enhance drug delivery, such as microneedles, ultrasound, and iontophoresis also for the treatment of diabetes (cluster 5); (iii) the development of TDD systems for a wider range of drug classes, including NSAIDs, pain management, biologics, and hormones (cluster 1, cluster 2). Some of the key technological trends reported in patents on TDD are (i) novel devices and methods to enhance drug delivery and achieve targeted drug release profiles; (ii) the use of TDD for new applications, such as the delivery of gene therapies and immunotherapies; the development of new manufacturing methods and technologies to improve the scalability and reproducibility of TDD systems.

Overall, the patent landscape tends to be more focused on specific products and applications, such as wearable devices and nanocarriers, to increase drug permeability and new formulations. In contrast, scientific research tends to be more exploratory and focused on developing a deeper understanding of the underlying science and mechanisms of transdermal drug delivery.

Bibliometric and patent results can be prospective indicators for the promising directions in the TDD field. The future landscape of TDD unfolds as a complex scenario, rich with opportunities and challenges that span diverse realms of research and development.

The quest for advanced materials and formulations not only seeks to improve drug permeation and skin penetration but also envisions the creation of novel, biocompatible materials capable of targeted and controlled release. Analogously, manufacturing innovations play a pivotal role in addressing the shifting trends in TDD methods. The research community is poised to delve into scalable and reproducible production techniques to keep pace with patent developments. This includes using innovative methods such as 3D printing for drug-loaded transdermal patches and automated assembly lines, ensuring both the quality and efficiency of TDD systems.

In the changing scientific field, a deeper understanding of barrier function and skin biology emerges as another critical way to optimize transdermal systems. By conducting molecular-level studies on how the skin responds to various formulations and delivery methods, researchers can unlock valuable insights. This understanding can be the key to developing more effective TDD solutions with better therapeutic results.

In general, non-invasive delivery technologies, such as microneedles, ultrasound, and iontophoresis, present exciting prospects for research. The exploration of other painless and patient-friendly alternatives to traditional methods stands as a promising continuation of the technological trends in TDD. In the same way, the integration of wearable devices with digital health platforms marks an interesting synergy. Smart, wearable TDD devices that offer real-time dose monitoring and regulation, integrated with IoT sensors and technologies, leveraging physical and digital knowledge, represent a dynamic approach to healthcare.

Expanding the scope of drugs suitable for transdermal delivery, especially biologics and hormones, is another emergent trend. Tackling challenges associated with transdermal biologics delivery, such as molecule size and skin barrier properties, becomes a focal point for ongoing research. This expansion aligns with the industry’s commitment to broadening the therapeutic applications of TDD. In particular, the application of TDD in gene therapies and immunotherapies introduces a novel research frontier. Designing vectors and carriers capable of traversing the skin barrier for the delivery of therapeutic genes or immunomodulators opens new possibilities, merging the realms of transdermal drug delivery and personalized medicine.

In the global health context, TDD assumes a crucial role in improving medication accessibility, particularly in low-resource settings. Research efforts focusing on the development of cost-effective, stable, and easy-to-use TDD systems with widespread distribution capabilities hold the potential to significantly impact global healthcare outcomes.

Finally, from an economic point of view, considering regulatory pathways and marketing strategies becomes crucial for the successful integration of TDD systems into traditional healthcare. Addressing the patent landscape, demonstrating clinical benefits, and ensuring cost-effectiveness serve as key considerations, driving the effective translation of TDD innovations from research to practical application.

## Figures and Tables

**Table 1:** The top 10 journals in publishing papers on TDD.

Rank	Source	IF (2022)	Total Publications (Percentage)	Total Citations
1	International Journal of Pharmaceutics	6.270	525 (6.7%)	23,596
2	Journal Of Controlled Release	11.467	262 (3.4%)	19,315
3	Journal Of Pharmaceutical Sciences	3.534	226 (2.9%)	6606
4	Drug Development and Industrial Pharmacy	3.225	178 (2.3%)	4365
5	AAPS Pharm SciTech	3.246	158 (2.0%)	4451
6	Journal Of Drug Delivery Science and Technology	3.981	155 (2.0%)	1837
7	Pharmaceutical Research	4.580	153 (2.0%)	7893
8	Pharmaceutics	6.072	135 (1.7%)	1724
9	European Journal of Pharmaceutics and Biopharmaceutics	5.589	126 (1.6%)	6486
10	Drug Delivery	6.420	122 (1.6%)	2462

**Table 2:** The top 10 highest cited articles on TDD.

Title	PY	Journal	Citations	Ref.
Biomedical applications of collagen	2001	International Journal of Pharmaceutics	1516	[18]
Penetration enhancers	2012	Advanced Drug Delivery Reviews	1509	[19]
Microneedles for transdermal drug delivery	2004	Advanced Drug Delivery Reviews	1102	[20]
Ethosomes—Novel vesicular carriers for enhanced delivery: Characterization and skin penetration properties	2000	Journal of Controlled Release	1044	[21]
Biodegradable polymer microneedles: Fabrication, mechanics, and transdermal drug delivery	2005	Journal of Controlled Release	693	[22]
Wearable/disposable sweat-based glucose monitoring device with multistage transdermal drug delivery module	2017	Science Advances	679	[23]
Microfabricated needles for transdermal delivery of macromolecules and nanoparticles: Fabrication methods and transport studies	2003	Proceedings of the National Academy of Sciences of the United States of America	658	[24]
Iontophoretic drug delivery	2004	Advanced Drug Delivery Reviews	641	[25]
Dissolving microneedles for transdermal drug delivery	2008	Biomaterials	639	[26]
Lipid vesicles and other colloids as drug carriers on the skin	2004	Advanced Drug Delivery Reviews	573	[27]

**Table 3:** The top 10 authors of papers on TDD.

Author Name	Country	Affiliation	Documents Count
Banga, A.K.	United States	University of Atlanta	78
Donnelly, R.F.	Ireland	Queen’s University Belfast	75
Prausnitz, M.R.	United States	Georgia Institute of Technology	69
Kalia, Y.N.	Suisse	Université de Genève	61
Fang, L.	China	Shenyang Pharmaceutical University	58
Mitragotri, S.	United States	Georgia Institute of Technology	50
Aqil, M.	Saudi Arabia	King Saud University	49
Stinchcomb, A.L.	United States	University of Maryland School of Pharmacy	46
Opanasopit, P.	Thailand	Silpakorn University	38
Shin, S.C.	South Korea	Chonnam National University	38

**Table 4:** Top 10 public funding sponsors in the TDD field.

Funding Sponsor	Country	Documents Count
National Natural Science Foundation	China	469
National Institutes of Health	United States	237
National Research Foundation	Korea	117
National Institute of Biomedical Imaging and Bioengineering	United States	79
Fundamental Research Funds for the Central Universities	China	74
Japan Society for the Promotion of Science	Japan	72
Conselho Nacional de Desenvolvimento Científico e Tecnológico	Brasil	70
Coordenação de Aperfeiçoamento de Pessoal de Nível Superior	Brasil	70
National Science Foundation	United States	70
Ministry of Education, Culture, Sports, Science and Technology	Japan	64

**Table 5:** Top 10 thematic areas on which TDD impacts.

Subject Area	Documents Count
Pharmacology, toxicology, and pharmaceutics	4778
Medicine	1790
Biochemistry, genetics, and molecular biology	1553
Chemistry	1337
Materials science	1132
Engineering	852
Chemical engineering	791
Physics	509
Immunology and microbiology	134
Multidisciplinary	124

**Table 6:** Clusters of the top 92 keywords occurring in papers on TDD, grouped in the main six clusters.

Keywords						
Cluster 1		Cluster 2	Cluster 3	Cluster 4	Cluster 5	Cluster 6
anti-inflammatory curcumin diclofenac sodium dissolving MNs ethosome hyaluronic acid hydrogel ibuprofen in vitro release indomethacin ketoprofen liposome meloxicam methotrexate microemulsion nanoemulsion nanoparticle	nanostructured lipid carriers oleic acid penetration enhancer percutaneous absorption psoriasis rheumatoid arthritis skin irritation skin penetration skin permeability skin permeation solid lipid nanoparticles stability stratum corneum sustained release topical delivery transdermal absorption transdermal permeation	bioavailability buprenorphine enhancer estradiol fentanyl human skin lidocaine microdialysis pain patch pharmacodynamics pharmacokinetic transdermal administration transdermal delivery system transdermal patch	absorption controlled release diclofenac diffusion drug delivery systems formulation hydrogels mathematical model percutaneous permeability protein delivery skin solubility surfactants	chitosan drug release flux hypertension in vitro in vivo nanoparticles permeation permeation enhancer release topical transdermal transdermal patches	diabetes drug delivery electroporation insulin iontophoresis microneedles sonophoresis ultrasound	ethosomes factorial design gel liposomes niosomes optimization proniosomes transfersomes

**Table 7:** Some examples of TDD systems, as reported in published articles.

Drug/Prodrug/NPs Released	Methods of Preparation and Formulation Details	System Operation	Inference	Cluster	Ref.
Ammonium glycyrrhizinate (A.G.)	Ammonium glycyrrhizinate—ultra-deformable liposomes (A.G.-ULs) were obtained by dissolving the drug in the lipid components during the synthesis of ULs (thin-layer evaporation technique)	High dimensional stable ULs pass intact through the skin and deliver AG in specific tissues in a controlled manner	Anti-inflammatory effect	6	[60]
Resveratrol/caffeic acid containing glycoconjugates	Imbibition of PEGDA/HEMA films in water solution containing the glycoconjugates	pH-dependent release of synthetic selenium-containing glycoconjugates	Antioxidant effect, potential wound healing acceleration	3	[61]
Horseradish peroxidase (HRP) enzyme	Silk MNs obtained by aqueous-based micro-molding and simultaneous loading with HRP	The degradation rate of silk fibroin and the diffusion rate of the entrained molecules can be controlled by adjusting post-processing conditions	Tunable release kinetics	5	[62]
Lidocaine hydrochloride (LIDH)	UV crosslinked methacrylated chondroitin sulfate (CS-MA) and polyvinylpyrrolidone (PVP) K29/32MNs loaded with LIDH	At body temperature, PVP K29/32 rapidly dissolves, with subsequent release of LIDH	Local anesthesia	5	[63]
Ibuprofenamine hydrochloride (2-(Diethylamino) ethyl 2-(4-isobutylphenyl) propionate hydrochloride	Clinical trial research, in which spray ibuprofenamine hydrochloride penetrates the skin and biological barrier into the lesion tissue after administration	Spray ibuprofenamine hydrochloride (prodrug of ibuprofen) penetrates the skin and is quickly converted into therapeutic ibuprofen	Anti-inflammatory effect	1	[33]
Ibuprofen	Hot-melt poly(ether-urethane)-silicone crosslinked pressure-sensitive adhesive (HMPSAs) drug reservoir formation	Adhesive matrix releases ibuprofen that penetrates the skin. The presence of chemical enhancers, di(ethylene) glycol monoethyl ether (DEGEE), facilitates the API penetration	Anaesthetic in case of moderate pain	1	[64]
Buprenorphine hydrochloride (Bup)	Electrospinning of poly (vinyl pyrrolidone) (PVP) and a blend of 50/50 *W*/*W* of buprenorphine-loaded poly(vinyl alcohol (PVA) and PVP polymer solutions in water, used as a drug carrier for buprenorphine (Bup): (Bup/PVP) and(Bup/PVP/PVA)	Bup-loaded crosslinked nanofibers improve carrier retention and provide a controlled release of Bup	Controlled release	2	[65]
Fentanyl citrate	Sucrose-based MN models are made from a water-soluble matrix premixed with fentanyl citrate	Dissolving MNs are submerged in a rectangular compartment. The top and the bottom of the compartment represent the SC and the bloodstream, respectively. Once an MN patch is applied to the skin, the needles penetrate the dermis and begin to dissolve.	Controlled release	2	[66]
Ampicillin sodium	Polyvinyl alcohol (PVA)/chitosan (CS) composite nanofibers are fabricated by electrospinning and then crosslinked through glutaraldehyde (GA)	Crosslinked PVA/CS composite nanofibers have a lower drug release rate and a smaller amount of drug burst release than that of PVA/CS, showing potential as TDD system	Controlled release of drugs	4	[67]
5-FU anticancer drug	5-fluorouracil-chitosan-carbon quantum dot-aptamer (5-FU-CS-CQD-Apt) nanoparticle is synthesized owing to W/O emulsification method	5-FU-CS-CQD-Apt shows a pH-sensitive and sustained drug release profile	Release of the drug in a controlled manner	4	[68]
Piroxicam	Nanoprecipitation technique is used for the preparation of drug-loaded Eudragit S100 (ES100)/NPs.	ES100 as a nanocarrier for transdermal delivery of Piroxicam	pH-sensitive permeation	3	[69]
Celecoxib (CXB)	CXB niosomes by thin film hydration method	The release of CXB from different niosomal gel formulations (Span 60 or Span 40 and cholesterol) depends on the viscosity of the prepared gels	Anti-inflammatory activity of the drug from niosomal gel formulations	1, 6	[70]

**Table 8:** Top 10 countries in which patents on TDD have been filed or granted.

Jurisdiction	Documents Count
United States	3002
China	793
European Patents	608
Canada	268
Republic of Korea	222
Australia	207
Japan	90
Mexico	84
Taiwan	68
United Kingdom	37

**Table 9:** TOP 10 Cooperative Patent Classification in the TDD field.

CPC	%
A61K9/0014	10.6
A61K9/7061	8.2
A61P29/00	6.0
A61M37/0015	5.8
A61K9/7084	5.8
A61P35/00	5.4
A61K47/10	5.3
A61K45/06	4.9
A61P43/00	4.7
A61P25/00	4.5

**Table 10:** Top 10 applicants in the TDD field.

Applicant Name	% Documents	Country
Noven Pharmaceutical Inc. (Miami, FL, USA)	4.3	USA
Alza Corp. (Mountain View, CA, USA)	3.1	USA
3M Innovative Properties Co. (Saint Paul, MI, USA)	2.0	USA
University of California	1.0	USA
Acrux Dds Pty LTD	1.0	Australia
Mylan Technologies Inc. (St. Albans, VT, USA)	0.9	USA
Kimberly-Clark Co. (Dallas, TX, USA)	0.9	USA
Corium International Inc. (Boston, MA, USA)	0.8	USA
Chrono Therapeutics Inc. (Hayward, CA, USA)	0.8	USA
Koninkl Philips Electronics Nv	0.7	Holland

**Table 11:** Top 10 cited active patents that refer to a medical device comprising a housing for TDD.

Application Number	Application Date	Title	Owners	Ref.
US 10 303 851 B2	2013/03/15	Physician-centric health care delivery platform	MD24 Patent Tech Llc	[77]
US 8 523 791 B2	2009/08/11	Multi-modal drug delivery system	CareWear Corp. (Reno, NV, USA)	[78]
US 9 375 529 B2	2009/09/02	Extended use medical device	Becton Dickinson and Company (Franklin Lakes, NJ, USA)	[79]
US 8 372 040 B2	2006/05/24	Portable drug delivery device including a detachable and replaceable administration or dosing element	Chrono Therapeutics Inc.	[80]
US 8 617 071 B2	2007/06/21	Analyte monitoring device and methods of use	Abbott Diabetes Care Inc. (Chicago, IL, USA)	[71]
US 8 252 321 B2	2007/10/31	Biosynchronous transdermal drug delivery for longevity, anti-aging, fatigue management, obesity, weight loss, weight management, delivery of nutraceuticals, and the treatment of hyperglycemia, Alzheimer’s disease, sleep disorders, Parkinson’s disease, aids, epilepsy, attention deficit disorder, nicotine addiction, cancer, headache and pain control, asthma, angina, hypertension, depression, cold, flu and the like	Chrono Therapeutics Inc.	[81]
US 7 658 728 B2	2006/01/10	Microneedle array, patch, and applicator for transdermal drug delivery	Yuzhakov Vadim V	[82]
US 9 186 372 B2	2013/05/21	Split dose administration	Moderna Therapeutics	[83]
US 2004/0116866 A1	2003/12/04	Skin attachment apparatus and method for patient infusion device	Insulet Corp	[84]
US 7 206 632 B2	2004/01/30	Patient sensory response evaluation for neuromodulation efficacy rating	Medtronic Inc.	[85]

**Table 12:** TOP 10 cited active patents that refer to TDD for the treatment of cancer.

Application Number	Application Date	Title	Owners	Note on TDD	Ref.
US 7 871 607 B2	2005/02/23	Soluble glycosaminoglycanases and methods of preparing and using soluble glycosaminoglycanases	Halozyme Inc.	Novel soluble neutral active hyaluronidase glycoproteins (sHASEGPs), methods of manufacture and administration (including TDD) that can be applied to enhance the bioavailability (and potentially improve other pharmacokinetic and/or pharmacodynamic properties) of pharmacologic and other agents that are useful for treating or diagnosing various disease conditions	[86]
US 7955597 B2	2002/10/26	Anti-il-6 antibodies, compositions, methods and uses	Centocor Inc.	Transdermal administration of anti-IL-6 antibody encapsulated in a delivery device such as a liposome or polymeric nanoparticles, microparticle, microcapsule, or microspheres (referred to collectively as microparticles unless otherwise stated)	[87]
US 11 154 559 B2	2012/09/28	Methods and compositions of bile acids	Gen Hospital Corp	A carrier that protects the composition against rapid release, such as a controlled release formulation, including implants, transdermal patches, and microencapsulated delivery systems	[88]
WO 2004/002417 A2	2003/06/27	Mammalian CH1 deleted mimetibodies, compositions, methods and uses	Centocor Inc.	CH1-deleted mimetibody or specified portion or variant in either the stable or preserved formulations or solutions described can be administered via a variety of delivery methods, including TDD	[89]
US 7 985 424 B2	2005/12/21	Dendritic polymers with enhanced amplification and interior functionality	Dendritic Nanotechnologies Inc.	Dendritic polymers can have utility in many applications (in vivo diagnostic imaging, drug delivery, drug discovery, in vitro diagnostics, coatings for medical devices, anti-biofouling coatings, TDD, chemotherapies, NIR absorbers, magnetic bioreactor, etc.)	[90]
WO 2005/032460 A2	2004/09/03	Human epo mimetic hinge core mimetibodies, compositions, methods and uses	Centocor Inc.	Mammalian EPO mimetic hinge core mimetibodies that can be used via contacting or administering by at least one mode comprising TDD	[91]
US 9 713 643 B2	2003/10/24	Cosmetic and pharmaceutical foam	Foamix Ltd.	Alcohol-free, pharmaceutical foam carrier and its use	[92]
US 7 591 806 B2	2005/05/18	High-aspect-ratio microdevices and methods for transdermal delivery and sampling of active substances	Xu Bai	High-aspect-ratio microdevices (including microneedles) and the method of making and using the same for TDD	[93]
WO 01/95935 A1	2001/01/22	Immunostimulatory nucleic acids for inducing a th2 immune response	Ottawa Health Research Inst	The compounds can be administered to the skin, e.g., topically in the form of skin cream, by injection, or any other method of administration where access to the skin cells and/or target APCs by the compounds is obtained	[94]
US 8 372 040 B2	2006/05/24	Portable drug delivery device including a detachable and replaceable administration or dosing element	Chrono Therapeutics Inc.	A device for TDD and administration of differing dosages at specific times of the day automatically pursuant to a pre-programmed dosage profile	[80]

**Table 13:** TOP 10 cited active patents that refer to TDD for the administration of prodrugs.

Application Number	Application Date	Title	Owners	Note	Ref.
US 7 871 607 B2	2005/02/23	Soluble glycosaminoglycanases and methods of preparing and using soluble glycosaminoglycanases	Halozyme Inc.	Novel soluble neutral active Hyaluronidase Glycoproteins (sHASEGPs), methods of manufacture and administration (including TDD) that can be applied to enhance the bioavailability (and potentially improve other pharmacokinetic and/or pharmacodynamic properties) of pharmacologic and other agents that are useful for treating or diagnosing various disease conditions	[86]
US 7 985 424 B2	2005/12/21	Dendritic polymers with enhanced amplification and interior functionality	Dendritic Nanotechnologies Inc.	Dendritic polymers can have utility in many applications (in vivo diagnostic imaging, drug delivery, drug discovery, in vitro diagnostics, coatings for medical devices, anti-biofouling coatings, TDD, chemotherapies, NIR absorbers, magnetic bioreactor, etc.)	[90]
WO2012118562A1	2012/03/02	Compositions and methods for treating depression, ADHD and other central nervous system disorders employing novel bupropion compounds, and methods for production and use of novel bupropion compounds and formulations	Rhine Pharmaceuticals Llc	Compositions and methods are disclosed using a purified (R)(-) enantiomer of bupropion (including prodrug) to treat central nervous system disorders	[95]
US 9 713 643 B2	2003/10/24	cosmetic and pharmaceutical foam	Foamix Ltd.	Alcohol-free, pharmaceutical foam carrier and its use	[92]
US 8 523 791 B2	2009/08/11	Multi-modal drug delivery system	Carewear Corp	A device for the transdermal delivery of a therapeutic agent at a treatment site comprising a mechanical vibration element, a light source, and a heating and/or cooling element	[78]
WO 01/95935 A1	2001/01/22	Immunostimulatory nucleic acids for inducing a th2 immune response	Ottawa Health Research Inst	The compounds can be administered to the skin, e.g., topically in the form of skin cream, by injection, or any other method of administration where access to the skin cells and/or target APCs by the compounds is obtained	[94]
US 10 369 204 B2	2009/10/02	Molecular vaccines for infectious disease	Agilent Technologies Inc.	Methods for construction of pharmamers, i.e., vaccine components characterized by their multimerization domain and the attached biologically active molecules, and their use in the preparation of vaccines that contain the pharmamers alone or in combination with other molecules	[96]
WO 2005/018530 A2	2004/08/20	Penetrating pharmaceutical foam	Foamix Ltd.	Alcohol-free cosmetic or pharmaceutical foam composition comprising water, a hydrophobic solvent, a surface-active agent, a gelling agent, an active component selected from the group of urea, hydroxy acid, and a therapeutic enhancer and a propellant. The foam further comprises active agents and excipients with therapeutic properties having enhanced skin penetration	[97]
US 8 668 937 B2	2012/03/17	Topical nitric oxide systems and methods of use thereof	Transdermal Biotechnology Inc.	Compositions for delivery of nitric oxide transdermally and/or to a mucosal surface	[98]
US 2017/0,232,115 A1	2012/10/12	Porous nanoparticle-supported lipid bilayers (protocells) for targeted delivery including transdermal delivery of cargo and methods thereof	Sandia Corp	The protocells enhance SC permeability and enable the transdermal delivery of active ingredients, including macromolecules	[99]

## Data Availability

Data are available on request.
